# A weighted fuzzy approach for the agility of sustainable product development process assessment: A case study in Chinese medium-sized enterprises

**DOI:** 10.1016/j.heliyon.2024.e34138

**Published:** 2024-07-04

**Authors:** Zhao Zhining, Hassan Alli, Masoud Ahmadipour, Rosalam Che me

**Affiliations:** a Faculty of Design and Architecture Universiti Putra Malaysia, 43400, UPM, Serdang, Selangor, Malaysia; b Institute of Power Engineering, Department of Electrical and Electronics Engineering, College of Engineering, Universiti Tenaga Nasional (UNITEN), 43000, Kajang, Selangor, Malaysia

**Keywords:** Agility, Sustainable product development, Fuzzy analytic hierarch process (FAHP), Fuzzy logic, Weighted fuzzy assessment method (WFAM)

## Abstract

The integration of sustainable practices within manufacturing organizations has become a necessity. However, ensuring a competitive edge in the market remains pivotal for the success of these sustainability initiatives. This research introduces an approach to harmonize the influence of sustainability and agility within the product development process, enabling enterprises to pursue sustainable manufacturing while upholding robust market competitiveness. The significance of this study lies in its combined utilization of expert insights and mathematical techniques to gauge the components and sub-components of sustainability and agility, thereby enhancing the precision of assessment outcomes. This accomplishment was achieved through the application of a Weighted Fuzzy Assessment Method (WFAM) for evaluating both product sustainability and agility. Employing the Fuzzy Analytic Hierarchy Process (FAHP), the study assigned weights to elements and sub-elements. Subsequently, employing fuzzy logic based on these derived weights, the study assessed the sustainability and agility scores in the product development process. Demonstrating the effectiveness of this devised methodology, the research employed a multi-functional electric bicycle as a case study. The outcomes highlight the potential the proposed method in attaining the varied objectives of sustainability and agility in product development.

## 1 Introduction

Sustainable development has become a prominent focus within industrial production and market dynamics, prompting widespread discussions in academic and industrial spheres [1]. With markets evolving constantly and new technologies emerging rapidly, future factories need to swiftly adapt their product development strategies, employ adaptable and efficient production methods, comply with increasing sustainability criteria, and navigate complex market requirements. Agility, characterized by cost competitiveness and flexibility, corresponds to the vested interests of enterprises aiming for sustainable development [2]. As a result, businesses spanning various industries are incorporating agile principles into their product development and innovation management practices [3,4].

Currently, the industry recognizes that the fundamental characteristic of sustainable products is their minimal environmental impact [[5], [6], [7], [8], [9]]. Ghadimi et al. [10] emphasized the significance of considering not only the sustainability of the final product but also the sustainability of the manufacturing processes involved in creating such products. To achieve sustainable product development, companies need to embrace practices like renewable energy usage, energy efficiency, adoption of green building principles, and the creation of other environmentally and socially equitable products. It is essential to concentrate on both the manufacturing process and the entire lifecycle of the product. Several design methodologies have been devised to improve processes, functionalities, and materials to enhance product sustainability while simultaneously cutting costs associated with production, disassembly, and recycling [11]. However, this hasn't effectively alleviated the market demand for sustainable product manufacturing. The crucial factor in securing market share continues to be the ability to respond swiftly to market demands. The traditional standardized mass-production approach has historically been successful in delivering goods quickly and at a low cost [12]. Nonetheless, it is no longer capable of meeting the evolving market requirements.

The ongoing progression of digitalization has led to shortened product life cycles, heightening the significance of time to market [13]. Additionally, motivated by the goal of enhancing customer benefits, there has been a recent surge in demand for personalized products [14]. In this dynamic business landscape, enterprises aim to achieve agility [15] and are integrating agile methodologies into their development processes [16]. Agile manufacturing was introduced to encourage companies to adopt flexible strategies across their organizations and improve responsiveness. Many agile methodologies originated from software development, among which Scrum stands out as one of the most recognized. Scrum revolves around principles such as transparency, review, and adaptation. Another widely used agile approach is design thinking, which encompasses various phases, each offering multiple development methods [17]. Design thinking focuses on understanding product users' needs, and swiftly generating concepts and prototypes based on this understanding. A more comprehensive approach to agile system design has been devised, covering product development, associated verification systems, and production systems. The core of this approach involves introducing specific agile components and integrating them into the existing framework of manufacturing companies.

However, sustainable product development is marked by its significant complexity, dynamism, and uncertainty [18]. This intricacy has led to an increased adoption of agile methodologies [19], especially in personalized application domains. Researchers have proposed frameworks for product development that integrate both sustainability and agility. For instance, Leong et al. recommended merging product management with agile methodologies in software development as a crucial step towards sustaining sustainability [20]. Žužek et al. [21] proposed that employing agile project management methods enhances the competitiveness of small and medium-sized enterprises in the manufacturing sector. Tripathi et al. [22] introduced an agile model that integrates lean, smart, and green innovation, targeting productivity improvement through optimized management systems. Shams et al. [23] introduced the concept of strategic agility, aiming for a comprehensive integration of agility across operational aspects encompassing production, information, technology, and the supply chain. Hariyani & Mishra [24] introduced the Integrated Sustainable Green Lean Six Sigma Agile Manufacturing System (ISGLSAMS). This framework extends the responsibilities of manufacturing organizations to include recycling and safe disposal of product, process, and supply chain waste. It also advocates for the adoption of a sustainable Rapid Response Manufacturing System to produce customized products sustainably. De Almeida et al. [25] applied the parallel evolutionary agile project management framework during the new product development process to expedite the final product delivery.

Numerous researchers argue that conducting a comprehensive analysis of sustainability and agility is crucial to underscore the significance of these factors. Albers et al. [26] propose a scenario- and requirement-based facilitation of agile transformation in product development, involving the identification and clustering of enterprise agile capabilities. Mathiyazhagan et al. [27] assert that integrating lean and agile practices can amplify sustainability. They utilize fuzzy set theory and best-worst methods to evaluate sustainable supply chain practices, offering managers insights to prioritize agile practices. Geyi et al. [28] through questionnaires, validate that agile capabilities are fundamental for optimizing sustainable practice outcomes. Vinodh & Devadasan [29] developed a measurement model for an agility index comprising 20 criteria, employing fuzzy logic. They establish an agility index based on data from a manufacturing organization, identifying barriers to achieving agility. This assessment method could assist enterprises in accelerating their agility. Vaishnavi & Suresh [30] conduct a readiness assessment for healthcare organizations to implement agility changes. Their conceptual model incorporates five enablers, twenty criteria, and fifty-six attributes, evaluating current readiness and advising management on corrective actions. Continuous assessment can elevate healthcare readiness for agile implementation. Vinodh et al. [31] use a scoring method to assess the agility of an Indian electric vehicle manufacturer, employing a multi-level fuzzy approach. They demonstrate that agility improvements are attainable through assessment efforts. Some researchers place agility within the economic dimension of sustainability, aiming to enhance overall enterprise capabilities through evaluation. Zarandi et al. [32] propose integrating an expert evaluation system to shorten material selection lists. This initial filtering can help identify suitable materials, increasing the likelihood of producing more sustainable products while saving time and cost. Wang et al. [33] introduce a multi-criteria decision-making model for optimizing supplier selection. This approach ranks potential suppliers, enhancing evaluation flexibility. Lu et al. [34] introduce a product design process model encompassing assessment streams—life cycle quality analysis, assessment, and cost—for functional, environmental, and economic evaluation. Zolfani et al. [35] argue that material selection for sustainable conceptual design doesn't require restructuring stages and procedures. They utilize multi-attribute decision-making methods to assign weights to sustainable material design standards, employing comparative research with SWARA and BWM methods.

Some existing approaches concentrate on either sustainability or agility, while a few attempt to address both simultaneously. However, a common issue across these methods is the lack of treating agility and sustainability as separate assessment dimensions, along with insufficient consideration for the weighting of elements and sub-elements. Notably, there's a scarcity of research that emphasizes continuous improvement, particularly in the realm of product development concerning agility and sustainability. This gap inhibits the attainment of more agile processes and sustainable products, which are crucial for overall enterprise sustainability. Therefore, the various important factors of sustainability and agility in the planning process of product development need to be ensured to be independent in the evaluation process based on adopting an integrated framework, to help enterprises improve the sustainability of products and the agility of the development process in a targeted and gradual manner, to enhance product competitiveness and meet the continuous development needs of enterprises.

Addressing these gaps found in previous studies, a new evaluation method is proposed to assess both product sustainability and agility. This novel approach combines fuzzy logic and the fuzzy analytic hierarchy process to deliver a comprehensive evaluation. The method employs the Fuzzy Analytic Hierarchy Process (FAHP) to assign precise weights to selected features and sub-features. Subsequently, utilizing these weights, fuzzy logic is used to gauge the sustainability and agility levels of the product. The Weighted Fuzzy Assessment Method (WFAM) assists decision-makers in achieving more informed decisions, ensuring a more accurate assessment through this proposed approach. Furthermore, this method introduces an enhancement to the improvement phase, making it more dynamic. Due to its dynamic nature derived from the stages of improvement, the recently introduced approach aims to aid decision-makers within manufacturing firms. Its purpose is to streamline decisions regarding subsequent actions that can elevate the sustainability of manufactured products and accelerate the accomplishment of developmental objectives. To demonstrate the novelty of this study, the main contributions are listed here:

✓ Integrating factors of sustainable design and agility provides enterprises with a product development strategy list that integrates agility and sustainability goals.
✓ A new multi-objective product development model combining sustainability and agility has been proposed. This new model decomposes the complexity of sustainable product development goals, enabling enterprises to complete product development in an efficient and low-cost way, and helping them adapt to dynamic market demands.
✓ Independently evaluating the sustainability and the agility level of the product development process provides a clearer display for decision-makers in the enterprise, to clarify the capability level and future improvement direction of the enterprise.
✓ Quantified the factors and indicators for achieving product sustainability and development process agility. This method facilitates communication and cooperation between different departments within the enterprise and is more conducive to communication and learning with external expert teams.

The remainder of this article is organized as follows: Section 2 delves into the mathematical fundamentals. Following that, Section 3 outlines the case study along with its outcomes. Sections 4, 5 explain comparative analysis and sensitivity analysis. Finally, Section 6 provides conclusions and a discussion of the discoveries.

## 2 Mathematical preliminaries

### 2.1 Fuzzy Analytic Hierarchy Process (FAHP)

Chang pioneered the extent analysis technique, commonly applied in addressing Fuzzy Analytic Hierarchy Process (FAHP) applications [36]. This method utilizes fuzzy numbers to measure the “extent,” generating fuzzy synthetic degree values for individual objects through the extent analysis process [37,38]. $X=\left\{x_{1},x_{2},\ldots ,x_{n}\right\}$ represents the elements or options within the alternatives as a set of distinct objects.

Moreover, the criteria elements are depicted as a collection of objectives, such as $U=\left\{u_{1},u_{2},\ldots ,u_{m}\right\}$. Adhering to Chang's extent analysis approach, every object undergoes individual extent analysis for each goal $g_{i}$ [36]. Consequently, Eq. (1) enables the derivation of m extent analysis values for each object.

(1) $$M_{g_{1}}^{1},M_{g_{2}}^{2},\ldots ,M_{g_{i}}^{m},\left(i=1,2,\ldots \;\ldots \;\ldots n\right)$$

The triangular fuzzy number $M_{g_{i}}^{j}$ is represented by (a,b,c). in the equation, where j=1,2,………m signifies triangular fuzzy numbers. Now, the procedure of Chang's extent analysis is outlined as [39]:

Step 1 The fuzzy synthetic extent value is specified as shown in Eq. (2).

(2) $$S_{i}=\sum_{j=1}^{m}M_{g_{i}}^{j}⨂{\left[\sum_{i=1}^{n}\sum_{j=1}^{m}M_{g_{i}}^{j}\right]}^{-1}$$

If $M_{g_{i}}^{j}=\left(a_{ij},b_{ij},c_{ij}\right),\text{then}\;\sum_{j=1}^{m}M_{g_{i}}^{j}$ using the fuzzy addition operation represents the m extent analysis values for a specific matrix as shown in Eq. (3).

(3) $$\sum_{j=1}^{m}M_{g_{i}}^{j}=\left(a_{i1},b_{i1},c_{i1}\right)⨁\left(a_{i2},b_{i2},c_{i2}\right)⨁\ldots ⨁\left(a_{im},b_{im},c_{im}\right)=\left(\sum_{j=1}^{m}a_{ij},\sum_{j=1}^{m}b_{ij},\sum_{j=1}^{m}c_{ij}\right)=\left(a_{i}^{\prime },b_{i}^{\prime },c_{i}^{\prime }\right)$$

To calculate ${\left[\sum_{i=1}^{n}\sum_{j=1}^{m}M_{g_{i}}^{j}\right]}^{-1}$, fuzzy addition needs to be executed as indicated in Eq. s (4) and (5).

(4) $$\sum \sum M_{g_{i}}^{j}=\sum_{i=1}^{n}\left(\sum_{j=1}^{m}a_{ij},\sum_{j=1}^{m}b_{ij},\sum_{j=1}^{m}c_{ij}\right)=\left(\sum_{i=1}^{n}a_{i}^{\prime },\sum_{i=1}^{n}b_{i}^{\prime },\sum_{i=1}^{n}c_{i}^{\prime }\right)$$

$$\begin{array}{c} {\left(\sum_{i=1}^{n}\sum_{j=1}^{m}M_{g_{i}}^{j}\right)}^{-1}=\left(1\backslash \backslash /\sum_{i=1}^{n}c_{i}^{\prime },1/\sum_{i=1}^{n}b_{i}^{\prime },1/\sum_{i=1}^{n}a_{i}^{\prime }\right) \end{array}$$

So:

(5) $$S_{i}=\sum_{j=1}^{m}M_{g_{i}}^{j}⨂{\left(\sum_{i=1}^{n}\sum_{j=1}^{m}M_{g_{i}}^{j}\right)}^{-1}=\left(a_{i}^{\prime },b_{i}^{\prime },c_{i}^{\prime }\right)⨂\left(1/\sum_{i=1}^{n}c_{i}^{\prime },1/\sum_{i=1}^{n}b_{i}^{\prime },1/\sum_{i=1}^{n}a_{i}^{\prime }\right)=\left(a_{i}^{\prime }/\sum_{i=1}^{n}c_{i}^{\prime },b_{i}^{\prime }/\sum_{i=1}^{n}b_{i}^{\prime },c_{i}^{\prime }/\sum_{i=1}^{n}a_{i}^{\prime }\right)=\left(a_{i},b_{i},c_{i}\right)$$

Step 2 Calculation of Possibility Degree: When: If $S_{i}=\left(a_{i},b_{i},c_{i}\right),S_{k}=\left(a_{k},b_{k},c_{k}\right)$, the possibility degree denoting $S_{i}\geq S_{k}$ that indicated by $V\left(S_{i}\geq S_{k}\right)$ is defined in Eq. (6).

(6) $$V\left(S_{i}\geq S_{k}\right)=\underset{y\geq x}{\sup}\left(\min\left\{\mu_{S_{i}}\left(x\right),\mu_{S_{k}}\left(y\right)\right\}\right)$$

It can also be expressed in an equivalent manner as Eq. s (7) and (8).

(7) $$V\left(S_{i}\geq S_{k}\right)=\text{hgt}\left(S_{i}\cap S_{k}\right)=\mu_{S_{i}}\left(d\right)$$

Then:

(8) $$\mu_{S_{i}}\left(d\right)=\left\{ \begin{array}{c} 1⟹\text{if}\;\left(a_{i}\geq a_{k}\right)\\ 0⟹\text{if}\;\left(a_{k}\geq c_{i}\right)\\ \frac{a_{k}-c_{i}}{\left(b_{i}-c_{i}\right)-\left(b_{k}-a_{k}\right)}⟹\text{otherwise} \end{array}\right.$$

where d represents the ordinate of the highest intersection point between $\mu_{S_{i}}\;\text{and}\;\mu_{S_{k}}$

Step 3 The possibility degree for a convex fuzzy number to exceed k convex fuzzy numbers $S_{i}$, where i=1,2,………k, can be defined by Eq. (9).

(9) $$V\left(S\geq S_{1},S_{2},\ldots ,S_{k}\right)=V\left(S\geq S_{1}\right),\left(S\geq S_{2}\right),\ldots ,\left(S\geq S_{k}\right)=\min\left(V\left(S\geq S_{1}\right),V\left(S\geq S_{2}\right),\ldots ,V\left(S\geq S_{k}\right)\right)=\min\;V\left(S\geq S_{i}\right)$$

If we assume that for $\left(k=1,2,\ldots \;\ldots \;\ldots n,k\neq i\right),d^{\prime }\left(A_{i}\right)=\min\;V\left(S_{i}\geq S_{k}\right)$ then, the weight vector is determined as shown in Eq. (10).

(10) $$W^{\prime }={\left(d^{\prime }\left(F_{1}\right),d^{\prime }\left(F_{2}\right),\ldots ,d^{\prime }\left(F_{n}\right)\right)}^{T}$$

where $F_{i}\left(i=1,2,\ldots \;\ldots \;\ldots n\right)\;\text{are}\;n$ elements.

Step 4 Through normalization, the normalized weight vectors are established as shown in Eq. (11).

(11) $$W={\left(d\left(F_{1}\right),d\left(F_{2}\right),\ldots ,d\left(F_{n}\right)\right)}^{T}$$

Where W represents a non-fuzzy number, providing the priority weights of one alternative over another.

### 2.2 Weighted fuzzy assessment method

**Fig. 1:**
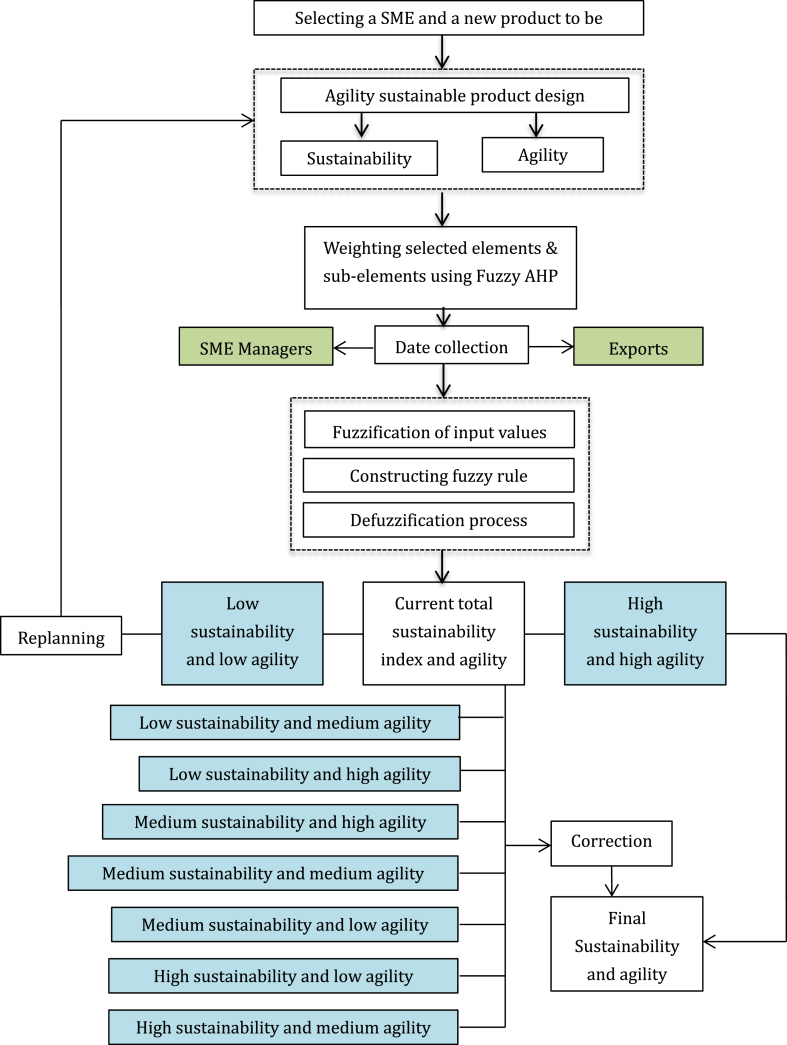
Framework of weighted fuzzy assessment method.

The methodology presented for this project is illustrated in Fig. 1. Its notable characteristic lies in its dynamic structure, enabling the case organization to embrace a more agile approach in creating sustainable products by drawing parallels from research. It encompasses a FAHP empowerment mechanism, an expert recommendation mechanism, and a fuzzy evaluation mechanism derived from insights provided by decision-makers’ expert both within and outside the organization. Elaborating on each step and conducting case studies are pivotal in clarifying these concepts. The proposed method comprises the following seven steps:

Step 1 Choose the subject matter experts (SMEs) and the products for assessment.

Select a product offered by a standard SME. This product is intended to foster the growth of said SME. Collect all the essential data required for analysis by scrutinizing the enterprise's systems and gathering necessary information from the provided data.

Step 2 Choose suitable variables and facilitators:

Incorporate the product development process into agile sustainable product design methodologies and identify all sustainability and agility variables along with their influencing factors. The validation of these research activities can be done by consulting decision-makers within enterprises.

Step 3 Assigning weights to the chosen sub-elements and influencing factors.

Compute the weights for all chosen sustainability elements and sub-elements utilizing FAHP. These weights will play a crucial role in step 5, facilitating the computation of scores for each element and sub-element. Incorporating these weights ensures a more precise and realistic evaluation, as they integrate the perspectives of enterprise decision-makers. Consequently, the overall sustainability index and agility index obtained in step 6 will be reliable, serving as a solid foundation for subsequent decision-making.

Step 4 Data collection

The data required can be sourced from the product development records of the chosen enterprise, focusing on agile and sustainable design methodologies through systematic analysis. It's important to note that the evaluation doesn't necessarily require the incorporation of all available data. The inclusion of data in the assessment is contingent upon the selection of specific elements and sub-elements deemed relevant to the evaluation process.

Step 5 Fuzzy evaluation

Fuzzy logic is applied for evaluating input data, where all clear data gathered in step 4 are transformed into membership levels representing linguistic terms in a fuzzy set. The significance and urgency of each variable are determined by business decision-makers, such as company owners, CEOs, general managers, or system managers. A target range or a specific value is designated for every input variable, indicating its minimum and maximum values. The selection of these reference values aligns with the manufacturing enterprise's objectives. Member functions for input variables are formulated based on these references. Subsequently, a linguistic value ranging from zero to one (0–1) is chosen as the benchmark for constructing the output membership function. A value of 0 signifies low sustainability and agility, while a value of 1 denotes high sustainability and agility. Upon establishing the membership functions for both input and output variables, a fuzzy rule base system is developed leveraging the insights and knowledge of decision-makers within the organization. The creation of rules for each sub-element is contingent on the count of input variables and the hierarchy of membership functions for these inputs, as determined by eq. (12) [40].

(12) $$R=n^{v}$$

The variable ' n' represents the quantity of grades within the membership function for input variables, ' v' denotes the count of input variables for each sub-element, and ' R' signifies the total number of rules to be created. Once the rules are constructed, the fuzzy inference system is deployed. During this stage, the outcome of each rule is generated by fuzzifying the inputs and passing them through the inference system. The output derived from the fuzzy inference system is then utilized as input for the defuzzification process. However, managing the rule evaluation and defuzzification of inputs becomes complex when dealing with a large volume of inputs and rules. To address this challenge, MATLAB can be employed to perform the fuzzy evaluation at this phase. Subsequently, scores for all sub-elements are calculated based on this fuzzy evaluation.

Step 6 Determine the sustainability and agility indices pertaining to the advancement of a product.

This phase involves computing the combined current sustainability index and agility index, which entails adding the weighted scores of both components, as outlined in eq. (14). Eq. (13) is utilized to determine the score for each individual sustainability element and agility element.

(13) $$I_{j}=\sum_{i}w_{ij}I_{ij}$$

where, $I_{j}$ is score of jth sustainability (agility) element. $w_{ij}$ is weight of ith sub sustainability (agility) element of jth sustainability (agility) element, element. $I_{ij}$ is score of ith sub sustainability (agility) element of jth sustainability (agility). i=1,2,………n is index of sub sustainability (agility) elements. j=1,2,………m is index of sustainability elements.

(14) $$I_{sustainability\;\left(agility\right)}=\sum_{i}w_{j}I_{j}$$

in which, $I_{j}$ is score of jth sustainability element. $w_{j}$ is weight of jth sustainability element. $I_{sustainability\;\left(agility\right)}$ is total sustainability index.

Using the determined index $I_{sustainability\;\left(agility\right)}$, decisions can be guided by predetermined thresholds. This process aims to ease the load on manufacturing managers by lessening their workload and the stress linked to endeavors aimed at improving their products. At this juncture, an array of decisions and suggestions regarding the system is presented to manufacturing or system managers, based on the results of the current sustainability (agility) index calculations. These suggestions might encompass conducting a thorough restructuring analysis or engaging in a review, rectification, and renewal assessment. The decisions and recommendations will largely align with expert opinions to a considerable degree.

Step 7 Compute the enhanced sustainability and agility indices for product development.

Implement the decisions formulated by experts in the prior stage onto the chosen product, re-assessing the product development procedure with a focus on sustainability and agility. Generate updated overall sustainability and agility indices based on this re-evaluation.

## 3 Case study and results

Comprehensive case studies regarding companies, products, data collection, sustainability, agility assessment for ASPDP, and ongoing design enhancements will be presented in this section.

Step 1 About the case study

An adaptable electric bike was selected for assessment [41] after discussions with business leaders and executives. The choice of a multifaceted electric bike stemmed from both market demand and competitive influences. Unanimously, managers and executives opted for this versatile electric bike as the focal product for the case study. The scope of analysis in this study spans the developmental phase of the product, encompassing the manufacturing process from design to evaluation. Hence, this case study specifically evaluates the product's developmental stage in its entirety.

Step 2 Criteria selection

The selection of components and sub-components in the weighted fuzzy assessment approach was established by referencing diverse research studies. Initially, these elements were derived from a comprehensive review of existing literature, after which discussions were held with decision-makers to refine them further. In this case study, the company owner, CEO, system manager and general manager were identified and involved as expert decision-makers. Their expertise was instrumental in validating the chosen sub-elements and factors that influence the assessment. Table 1 provides a comprehensive overview of all the selected elements and sub-elements.

Step 3 Assigning weights to chosen variables and facilitators through the employment of FAHP.

**Table 1:** Selected elements and sub elements of sustainability.

Index (level zero)	Elements (level one)	Sub elements (level two)	Reference
Sustainability	Product Technology	Information and communications technology (ICT)	[[42], [43], [44]]
		Additive technology (AT)	
	Sustainable Design Methods	Modular Design (MD)	[[45], [46], [47], [48], [49]]
		Computer-aided design (CAD)	
		Systemic design methodology (SDM)	
		Design for longevity (DL)	
	Product Material	Low impact materials (LIM)	[50]
	Product Performance	Cost benefit analysis (CBA)	[51]
	Product Safety	Source credibility (SC)	[[52], [53], [54]]
		Warranty (Warr)	
		Guide by policies (GP)	
Agility	Time-based Manufacturing	Customer orientation (CO)	[[55], [56], [57]]
		Formalization (Fo)	
		Product family architecture (PFA)	
	Flexible Process	Collaboration inside of organisation (CIO)	[58]
		Production focuses on flexible customer needs (PFFC)	
	Integrated Innovation	Cooperative relationship (COOP-R)	[59]
		Commitment relationship (COMM-R)	

During this phase, the elements and sub-elements related to product sustainability and agility undergo weighting using FAHP. As depicted in Table 1, a hierarchical structure with three levels has been established. Level zero signifies the primary objective, namely, the product sustainability and agility index. Level one comprises the elements utilized for assessing product sustainability and agility, while level two delineates the sub-elements used in the evaluation process. In this study, to streamline the fuzzy logic evaluation process, the influencing factors were not subjected to weighting based on the decision makers' opinions; hence, these factors were excluded from the FAHP process. At this stage, a panel of four experts collaborated to conduct pairwise comparisons regarding the relative significance of elements and sub-elements using the fuzzy scale outlined in Table 2. Initially, experts compared the core factors with an overarching sustainability index, following which they compared the sub-elements with the principal elements. Table 3, Table 4, Table 5, Table 6 display the outcomes of such comparisons, using product technology and sustainable design methods as exemplars. Subsequently, in line with Chang's FAHP methodology, weights were assigned to both elements and sub-elements. The resulting weights for these elements and sub-elements are presented in Table 7.

Step 4 Data collection

**Table 2:** The linguistic variables and the corresponding fuzzy numbers.

Linguistic scale	Triangular fuzzy number
Just equal	(1,1,1)
Equally important	(2/3,1,3/2)
Weakly important	(1,3/2,2)
Strongly more important	(3/2,2,5/2)
Very strong more important	(2,5/2,3)
Absolutely more important	(5/2,3,7/2)

**Table 3:** Fuzzy pairwise comparison matrixes for sustainability elements.

	Product Technology	Sustainable Design Methods	Product Material	Product Performance	Product Safety
Product Technology	(1,1,1)	(3/2,2,5/2)	(2/3,1,3/2)	(1,3/2,2)	(2/3,1,3/2)
Sustainable Design Methods	(2/5,1/2,2/3)	(1,1,1)	(2/3,1,3/2)	(2/3,1,3/2)	(1/3,2/5,1/2)
Product Material	(2/3,1,3/2)	(2/3,1,3/2)	(1,1,1)	(1/2,2/3,1)	(2/5,1/2,2/3)
Product Performance	(1/2,2/3,1)	(2/3,1,3/2)	(1,3/2,2)	(1,1,1)	(1/2,2/3,1)
Product Safety	(2/3,1,3/2)	(2,5/2,3)	(3/2,2,5/2)	(1,3/2,2)	(1,1,1)

**Table 4:** Fuzzy pairwise comparison matrixes for agility elements.

	Time-based Manufacturing	Flexible Process	Integrated Innovation
Time-based Manufacturing	(1,1,1)	(1,3/2,2)	(2/3,1,3/2)
Flexible Process	(1/2,2/3,1)	(1,1,1)	(2/3,1,3/2)
Integrated Innovation	(2/3,1,3/2)	(2/3,1,3/2)	(1,1,1)

**Table 5:** Fuzzy pairwise comparison matrixes for sustainability sub elements.

	Information and communications technology	Additive technology
Information and communications technology	(1,1,1)	(2/3,1,3/2)
Additive technology	(2/3,1,3/2)	(1,1,1)

**Table 6:** Fuzzy pairwise comparison matrixes for sustainability sub elements.

	Modular Design	Computer-aided design	Systemic design methodology	Design for longevity
Modular Design	(1,1,1)	(2/3,1,3/2)	(1/2,2/3,1)	(1/2,2/3,1)
Computer-aided design	(2/3,1,3/2)	(1,1,1)	(1,3/2,2)	(2/3,1,3/2)
Systemic design methodology	(1,3/2,2)	(1/2,2/3,1)	(1,1,1)	(2/3,1,3/2)
Design for longevity	(1,3/2,2)	(2/3,1,3/2)	(2/3,1,3/2)	(1,1,1)

**Table 7:** Results of the elements and sub elements weights.

Index	Elements	Weight	Sub elements	Weight
Sustainability	Product Technology	0.27	ICT	0.50
			AT	0.50
	Sustainable Design Methods	0.10	MD	0.20
			CAD)	0.27
			SDM	0.25
			DL	0.27
	Product Material	0.13	LIM	1
	Product Performance	0.17	CBA	1
	Product Safety	0.33	SC	0.23
			Warr	0.38
			GP	0.38
Agility	Time-based Manufacturing	0.38	CO	0.38
			Fo	0.33
			PFA	0.28
	Flexible Process	0.29	CIO	0.50
			PFFC	0.50
	Integrated Innovation	0.33	COOP-R	0.50
			COMM-R	0.50

### 3.1 Sustainability element data

#### 3.1.1 Product technology element data

Product technology comprises two main components: information and communication technology along with additive technology. Information and communication technology involves managing standardized data throughout a product's lifespan, facilitating data exchange between computer systems within and across enterprises, and aiding in the flow of product information within the supply chain. Within this research initiative, information and communication technologies are employed to seamlessly integrate vital information concerning the materials utilized in a product. This integration allows for access to and utilization of this information at any stage of the product life cycle, ranging from the design phase to disposal. Embracing these technologies enables companies to enhance the environmental sustainability of their e-bike development processes, prompting a comprehensive evaluation of the true environmental impact of their products or services throughout the entire life cycle. Additionally, the company intends to utilize additive technology to produce certain components, resulting in cost and time savings during the development of new electric bicycles. This approach not only aids in lightweighting but also significantly bolsters the endurance of electric bicycles.

#### 3.1.2 Sustainable design methods element data

Sustainable design methodologies encompass four key aspects: Modular Design, Computer-Aided Design (CAD), Systemic Design Methodology, and Design for Longevity. Modular Design involves the creation of self-contained units or modules, each fulfilling a specific function, aiming to break down complex systems into manageable, interchangeable, and scalable components. This approach is pivotal in developing a versatile electric bicycle that meets various usage scenarios and young people's diverse needs. By modularizing the bicycle, users can freely assemble and match functions to align with their preferences and habits. Systemic Design Methodology tackles complex problems by considering the interconnectedness and interdependence of elements within a system. Employing this method early in the development of multifunctional electric bicycles is crucial. Designers need holistic thinking and interdisciplinary collaboration, iterating designs based on feedback to achieve optimal product excellence. Design for Longevity emphasizes creating products with extended lifespans and durability, addressing the environmental impact of frequent product replacements and disposals. This focus on durability aligns with sustainable development goals by reducing waste. Utilizing Computer-Aided Design (CAD) is essential in this product's development, ensuring precision, accuracy, and efficiency in design. Digital modeling allows for swift modifications and iterations during the design process, facilitates design visualization, solution simulation, and analysis, streamlines team collaboration, and simplifies document management.

#### 3.1.3 Product element data

The primary focus in selecting materials for the product centers on low impact materials, which are those that exhibit a reduced environmental and ecological impact when compared to conventional materials. The company's development of this electric bicycle emphasizes the importance of materials that are recyclable, durable, locally obtained, and conducive to waste reduction. As an illustration, the electric bicycles under development will be equipped with graphene batteries known for their fast-charging capabilities, extended battery life, enhanced safety features, and repairability.

#### 3.1.4 Product performance element data

The primary approach adopted by the company for evaluating Product Performance is through Cost benefit analysis. This methodology involves assessing the economic viability of a project by juxtaposing overall costs against total benefits. This analytical process aids decision-makers in determining the economic justification of product development and whether the benefits surpass the associated costs. In this context, the costs incurred in the self-development of this electric product encompass research and development expenditures, operational expenses, equipment maintenance, and any other relevant costs. Conversely, the benefits encompass the anticipated revenue generated by the new product, cost reductions, enhancements in certain component technologies, and similar gains.

#### 3.1.5 Product safety element data

Product Safety encompasses three key elements: source credibility, Warranty, and guide by policies. Credible Sourcing denotes the company's commitment to maintaining integrity, transparency, and objectivity in disclosing product information, thereby enhancing the company's reputation. The company will offer warranty services to consumers. Regarding policy regulations, the company prioritizes leveraging government tax credits and research and development (R&D) funding for e-bike development. Simultaneously, adherence to environmental standards in battery production, compliance with import-export regulations, and adherence to recycling and disposal policies are essential aspects for the company to consider.

### 3.2 Agility element data

#### 3.2.1 Time-based manufacturing element data

Time-based Manufacturing comprises three key elements: Customer orientation, formalization, and product family architecture. Customer orientation pertains to a company's strategic mindset prioritizing meeting customer needs and expectations, aiming to create value and long-term relationships. In the creation of a versatile electric bicycle, designers actively engaged with customer feedback, continuously improving based on insights, and committing to delivering an exceptional customer experience. For instance, recognizing the trend of younger people using electric bicycles for short-distance travel and seeking more entertainment, designers incorporated scooter features into the electric bicycle. This not only boosted its style but also made it suitable for various entertainment scenarios. Formalization involves establishing and upholding formal structures, procedures, rules, and documentation within an organization. To ensure efficient management and collaboration, product development embraced a standardized process, enhancing consistency, reliability, predictability, and control. This fosters a clear organizational structure, delineates roles and responsibilities, and ensures systematic and unified work processes. Product family architecture refers to the strategic design of related products within a company's portfolio. The aim here is to broaden the scope of multi-functional electric bicycles by creating core components during the development process that are versatile and adaptable for different product variants or versions. This approach streamlines product variability management, reduces development time and costs by leveraging existing components and design elements. Ultimately, this simplified model encompasses design, manufacturing, maintenance, and disposal, aiming for sustainability, cost-effectiveness, and better responsiveness to diverse market needs.

#### 3.2.2 Flexible process element data

The Flexible Process is composed of two main components: Collaboration inside of organisation and production focuses on flexible customer needs. Collaboration inside of organisation refers to the coordinated effort of individuals, teams, or departments working together to achieve shared objectives, resolve issues, and improve overall organizational effectiveness. In the context of product development, diverse teams from various disciplines collaborate by sharing information, resources, and expertise, relying on a culture that fosters teamwork and cooperation. Therefore, the company encourages open and transparent communication, facilitates the establishment of strong interpersonal bonds among team members, sets common goals for all, and adopts adaptable work arrangements. Production focuses on flexible customer needs underscores the capacity of production systems to adapt, customize, and modify their output based on the specific desires and preferences of customers. This adaptability is a critical factor enabling enterprises to thrive amidst intense market competition. Consequently, the company has been implementing product diversification strategies, giving weight to customer feedback, emphasizing the efficiency and promptness in delivering products to customers, employing advanced technologies such as automation, data analysis, and real-time monitoring to bolster the flexibility and responsiveness of the production process. Additionally, it emphasizes collaboration with suppliers and supply chains, aiming for seamless material and information flow. Moreover, it continuously enhances production systems, aiming to identify and implement efficiencies and innovations that align with evolving customer needs.

#### 3.2.3 Integrated innovation element data

Integrated Innovation comprises two primary aspects: cooperative relationship and commitment relationship. Cooperative relationship signifies the cooperative and mutually advantageous association between enterprises. Within this partnership, involved parties collaborate towards shared objectives, pooling resources and aiding each other to enhance the collective success or prosperity. In this project, certain component developments necessitate collaboration with other companies. Additionally, activities related to product sales, services, and promotional initiatives are achieved through collaborations between various enterprises. Hence, the company has forged strong mutually beneficial ties with both upstream and downstream companies in the supply chain. It upholds effective communication, mutual trust, and is dedicated to resolving issues in a manner that preserves the overarching collaborative efforts in partnerships. A commitment relationship denotes a sense of dedication, loyalty, and responsibility between businesses. For partners engaged in enduring relationships, the company is committed to nurturing and preserving these connections, investing time, effort, and resources to foster and sustain these associations.

Step 5 Fuzzy evaluation

During this phase, explicit input and output variables are transformed into membership degrees using fuzzy set terminology, as detailed in Table 8, Table 9. Following this, a fuzzy rule base is established utilizing fuzzy variables, incorporating membership levels for clear input and output variables determined through consultations with policymakers and an extensive literature review. The decision was made to categorize input variable levels as low, medium, and high. Similarly, output variable membership levels were defined as low, low to medium, medium, medium to high, and high. Additionally, the MATLAB fuzzy logic package was employed in step 5 of the process. In this stage, MATLAB was utilized to adjust the parameters of the membership function and formulate certain rules. Equation (12) was utilized to calculate the number of rules to construct for each specific element. Drawing from the knowledge of the four decision makers, realistic rules were defined for the rule base. Moreover, to ensure precise and unequivocal evaluation, the entire knowledge was translated into rules, with Table 10 displaying some of the rules within the rule base.

Step 6 Compute the index for sustainability and agility in product development.

**Table 8:** Sustainability sub-elements and their membership functions.

Linguistics value	Numerical range	Linguistics value	Numerical range	Linguistics value	Numerical range
Linguistics value: ICT		Linguistics value: SDM		Linguistics value: SC	
Low	[0 1 2]	Low	[0 1 2]	Low	[0 1 2]
Medium	[1 2 3]	Medium	[1 2 3]	Medium	[1 2 3]
High	[2 3 4]	High	[2 3 4]	High	[2 3 4]
Linguistics value: AT		Linguistics value: DL		Linguistics value: Warr	
Low	[0 1 2]	Low	[0 1 2]	Low	[0 1 2]
Medium	[1 2 3]	Medium	[1 2 3]	Medium	[1 2 3]
High	[2 3 4]	High	[2 3 4]	High	[2 3 4]
Linguistics value: MD		Linguistics value: LIM		Linguistics value: GP	
Low	[0 1 2]	Low	[0 1 2]	Low	[0 1 2]
Medium	[1 2 3]	Medium	[1 2 3]	Medium	[1 2 3]
High	[2 3 4]	High	[2 3 4]	High	[2 3 4]
Linguistics value: CAD		Linguistics value: CBA			
Low	[0 1 2]	Low	[0 1 2]		
Medium	[1 2 3]	Medium	[1 2 3]		
High	[2 3 4]	High	[2 3 4]

**Table 9:** Agility sub elements and their membership functions.

Linguistics value	Numerical range	Linguistics value	Numerical range	Linguistics value	Numerical range
Linguistics value: CO		Linguistics value: CIO		Linguistics value: COOP-R	
Low	[0 1 2]	Low	[0 1 2]	Low	[0 1 2]
Medium	[1 2 3]	Medium	[1 2 3]	Medium	[1 2 3]
High	[2 3 4]	High	[2 3 4]	High	[2 3 4]
Linguistics value: Fo		Linguistics value: PFFC		Linguistics value: COMM-R	
Low	[0 1 2]	Low	[0 1 2]	Low	[0 1 2]
Medium	[1 2 3]	Medium	[1 2 3]	Medium	[1 2 3]
High	[2 3 4]	High	[2 3 4]	High	[2 3 4]
Linguistics value: PFA					
Low	[0 1 2]				
Medium	[1 2 3]				
High	[2 3 4]

**Table 10:** Some rule examples from the rule base.

Rule no.	Rules
Rule 1	If the levels of Information and Communications Technology and Additive Technology are both high, then the resulting Product Technology score will also be high.
Rule 2	If the degree of Modular Design is moderate, Computer-aided design is high, Systemic design methodology is moderate, and Design for longevity is low, the resultant score for Sustainable Design Methods will be moderate.
Rule 3	If there is low source credibility, alongside moderate levels of warranty and guide by policies, the resulting score for Sustainable Design Methods would range from medium to low.
Rule 4	If the level of Collaboration inside of organisation is moderate, coupled with low emphasis on production focuses on flexible customer needs, the resulting score for Integrated Innovation would range from medium to low.

The scores for individual sub-elements of sustainability and agility are computed, and their overall sustainability scores are derived using equation (13). Table 11, Table 12 exhibit the scores pertaining to selected sub-elements within each sustainability and agility component. Subsequently, upon obtaining all sub-scores for sustainability and agility, the overall Sustainability and Agility Index for the electric bicycle is calculated employing Equation (14), delineated in Table 11, Table 12 as well.

**Table 11:** Overall sustainability scores of current designs.

Element	Sub element	Unweighted sub element score	Weighted	Element score	Weight element score
Product Technology	ICT	0.56	0.34	0.52	0.12
	AT	0.48	0.18		
Sustainable Design Methods	MD	0.48	0.12	0.62	0.14
	CAD	0.64	0.12		
	SDM	0.77	0.10		
	DL	0.56	0.10		
Product Material	LIM	0.48	0.48	0.48	0.10
Product Performance	CBA	0.48	0.48	0.48	0.10
Product Safety	SC	0.41	0.24	0.54	0.11
	Warr	0.58	0.16		
	GP	0.52	0.14		
**Total Sustainability Index:**					**0.57**

**Table 12:** Overall agility scores of current designs.

Element	Sub element	Unweighted sub element score	Weighted	Element score	Weight element score
Time-based Manufacturing	CO	0.51	0.18	0.56	0.20
	Fo	0.30	0.22		
	PFA	0.58	0.16		
Flexible Process	CIO	0.52	0.22	0.48	0.16
	PFFC	0.64	0.26		
Integrated Innovation	COOP-R	0.58	0.30	0.52	0.16
	COMM-R	0.73	0.22		
**Total Agility Index:**					**0.52**

For design application, three distinct ranges have been defined. In this study, the sustainability (or agility) index ranged between 0 and 1. If the index falls within 0–0.33, it indicates a low level of sustainability (or agility). An index within 0.34–0.66 is considered indicative of a moderate level of sustainability (or agility), while an index ranging from 0.67 to 1 suggests a high level of sustainability (or agility). Within each of these defined scopes, discussions can be initiated with enterprise managers. For instance, the development of products with low sustainability would necessitate an extensive overhaul of the product plan, involving significant adjustments based on factory limitations. Similarly, planning for products with low agility requires modifications in variables that have negative impacts. The outcomes from this phase serve as an expert system, elucidating the weaknesses in current product development to enterprise management personnel.

In the ongoing phase, the current sustainability assessment of the product indicates a “moderate sustainability” level. As a result, there exists an opportunity to enhance the product's sustainability. Decision-makers have the ability to implement various measures such as redesigns, revisions, updated research, material substitutions, etc., aimed at elevating the sustainability level within the system. These actions are intended to tackle the identified weaknesses in the product design. To pinpoint these weaknesses accurately, the scores obtained for the 11 sustainability sub-elements form the basis, and they are multiplied by the weights acquired in step 3. These resultant figures are also showcased in Table 11 within the “Unweighted” column, denoted as “Sub-element score.” Significantly, among the various sub-elements, the scores for product materials and product performance emerge as the lowest. This outcome may serve as guidance to product designers or be considered in product decision-making, potentially influenced by the higher costs associated with low-impact materials that designers might overlook. There's a need for further investigation to explore the substitution of non-renewable materials with renewable alternatives, or at least consider non-renewable materials that offer advantages such as reduced manufacturing costs, lighter weight, fewer manufacturing processes, etc.

Simultaneously, referencing Table 12 reveals that the existing agility of the product has been identified as “moderately agile.” Hence, there remains the potential for the system to achieve a higher level of agility, allowing decision-makers to implement updates in development processes, management practices, and collaboration efforts to enhance the agility level.

In this study, the company's objective is to incorporate lighter and more durable exterior materials with the strategic aim of reducing the overall product weight, a potentially significant achievement. Consequently, another sustainability assessment will be necessary to showcase the impact of these alterations on the product's overall sustainability index. Concurrently, the company aims to improve process formalization to enhance internal collaboration efficiency to some extent. Moreover, business managers have suggested reducing product development time by expanding collaborations involving additional product components. Another assessment of agility will illustrate changes in the overall agility index resulting from these initiatives.

Step 7 Enhance the index for sustainability and agility in product development.

Expert assessments provide novel perspectives on potential modifications in sustainability and agility. In this fresh evaluation, these alterations are taken into consideration, resulting in the computation of revised weighted scores and an improved overall sustainability and agility index. The panel of experts utilized fuzzy scales to assess the relative significance of variable and driver pairs. It is imperative for the expert group to factor in the developmental experience of business managers and elevate the levels of sustainability and agility. Initially, the experts conducted comparisons between the variables and the overall sustainability index, followed by a comparison between the drivers and the variables. The outcomes of these comparisons are outlined in Table 13 and Table 14.

**Table 13:** Sustainability score after replacement.

Element	Sub element	weighted sub element score	Element score	Weight element score
Product Technology	ICT	0.28	0.58	0.12
	AT	0.30		
Sustainable Design Methods	MD	0.20	0.64	0.15
	CAD	0.18		
	SDM	0.12		
	DL	0.14		
Product Material	LIM	0.52	0.52	0.12
Product Performance	CBA	0.52	0.52	0.10
Product Safety	SC	0.18	0.56	0.12
	Warr	0.22		
	GP	0.16		
**Total Sustainability Index:**				**0.61**

**Table 14:** Overall score of agility by experts.

Element	Sub element	weighted sub element score	Element score	Weight element score
Time-based Manufacturing	CO	0.22	0.58	0.22
	Fo	0.18		
	PFA	0.18		
Flexible Process	Collaboration	0.30	0.55	0.18
	PFFC	0.25		
Integrated Innovation	COOP-R	0.32	0.52	0.16
	COMM-R	0.20		
**Total Agility Index:**				**0.56**

## 4 Competitive analysis

It is imperative for the expert group to factor in the developmental experience of business managers and elevate the levels of sustainability and agility. To evaluate the effectiveness of the proposed method to gauge the components and sub-components of sustainability and agility, it is replaced by two well-known algorithms namely, interval-valued hesitant fuzzy set (IVHFE) [60], and an IVIF-ELECTRE outranking method [61] in the proposed strategy and their obtained results are compared in Table 15, Table 16. For a fair comparison, all mentioned methods in Table 15, Table 16 have the same candidate inputs and strategy. As seen, the proposed method results in the highest total Sustainability index and total agility index among all methods.

**Table 15:** Comparison of sustainability score after replacement by different methods.

Element	Different algorithms											
	IVIF-ELECTRIC				IVHFE				The proposed method			
	SE	WSES	ES	WES	SE	WSES	ES	WES	SE	WSES	ES	WES
Product Technology	ICT	0.26	0.54	0.12	ICT	0.28	0.58	0.12	ICT	0.28	0.58	0.12
	AT	0.28			AT	0.30			AT	0.30		
Sustainable Design Methods	MD	0.22	0.72	0.18	MD	0.22	0.66	0.15	MD	0.20	0.64	0.15
	CAD	0.16			CAD	0.18			CAD	0.18		
	SDM	0.14			SDM	0.12			SDM	0.12		
	DL	0.16			DL	0.14			DL	0.14		
Product Material	LIM	0.48	0.48	0.08	LIM	0.50	0.50	0.10	LIM	0.52	0.52	0.12
Product Performance	CBA	0.48	0.48	0.08	CBA	0.54	0.54	0.10	CBA	0.52	0.52	0.10
Product Safety	SC	0.20	0.58	0.12	SC	0.18	0.56	0.12	SC	0.18	0.56	0.12
	Warr	0.24			Warr	0.22			Warr	0.22		
	GP	0.14			GP	0.16			GP	0.16		
**Total Sustainability Index:**	0.58				0.59				**0.61**			
SE: Sub-element; WSES: weighted sub-element score; ES: Element score; WES: Weight element score.

**Table 16:** Comparison of overall score of agility by experts by different methods.

Element	Different algorithms											
	IVIF-ELECTRIC				IVHFE				The proposed method			
	SE	WSES	ES	WES	SE	WSES	ES	WES	SE	WSES	ES	WES
Time-based Manufacturing	CO	0.20	0.56	0.20	CO	0.22	0.58	0.22	CO	0.22	0.58	0.22
	Fo	0.20			Fo	0.18			Fo	0.18		
	PFA	0.16			PFA	0.18			PFA	0.18		
Flexible Process	Collaboration	0.30	0.55	0.18	Collaboration	0.30	0.58	0.18	Collaboration	0.30	0.55	0.18
	PFFC	0.25			PFFC	0.28			PFFC	0.25		
Integrated Innovation	COOP-R	0.28	0.52	0.16	COOP-R	0.30	0.52	0.16	COOP-R	0.32	0.52	0.16
	COMM-R	0.24			COMM-R	0.22			COMM-R	0.20		
**Total Agility Index**	0.54				0.56				**0.56**			
SE: Sub-element; WSES: weighted sub-element score; ES: Element score; WES: Weight element score.												

## 5 Sensitivity analysis

The robustness of the decisions presented was examined through a one-at-a-time sensitivity analysis. This involved systematically adjusting the weight of each criterion from 0 to 1 in increments of 0.1 and recalculating the alternative scores accordingly. In this analysis, when the weight of one criterion was altered, the weights of the remaining criteria were adjusted proportionally to their initial importance. As a result, the total weight of all criteria remained constant at 1 in every scenario.

First, the relationship between alternatives and criteria is shown in Table 17. Changes in C7 (Flexible Process) can influence the rankings of alternatives of A12- A18, which consistently remains the least favorable option. C6 (Time-based Manufacturing) and C8 (Integrated Innovation) are also influential criteria that significantly impact alternative rankings. Conversely, C1 (Product Technology) and C5 (Product Safety) have a lesser effect on the rankings of alternatives. Notably, A15 (Collaboration inside of organization), the poorest alternative, remains unaffected and consistently ranks lowest in all scenarios. Interestingly, the positions of A5 (Systemic design methodology) and A11 (Guide by policies) can be interchanged by adjusting the weight of each criterion. Critical breakpoints for the weight of C7 include values such as 0.5, 0.7, 0.8, and 0.9. For instance, when the weight of C7 is 0.5, the top three rankings shift to A6 > A1 > A10 instead of A6 > A10 > A1. If C7 were the sole criterion, with a weight of 1.00, the new ranking of alternatives would be A10 > A6 > A7 > A1 > A4 > A5 > A11 > A2 > A3 > A8 > A9>A12 > A14 > A13 > A16 > A18 > A17 > A15, with A15 consistently identified as the least desirable option.

**Table 17:** Alternatives and criteria.

Alternative		Criteria	
A_1_	ICT	C_1_	Product Technology
A_2_	AT	C_2_	Sustainable Design Methods
A_3_	MD	C_3_	Product Material
A_4_	CAD)	C_4_	Product Performance
A_5_	SDM	C_5_	Product Safety
A_6_	DL	C_6_	Time-based Manufacturing
A_7_	LIM	C_7_	Flexible Process
A_8_	CBA	C_8_	Integrated Innovation
A_9_	SC		
A_10_	Warr		
A_11_	GP		
A_12_	CO		
A_13_	Fo		
A_14_	PFA		
A_15_	CIO		
A_16_	PFFC		
A_17_	COOP-R		
A_18_	COMM-R		

Regardless of changes in other criteria, A6 emerges as the preferred choice across variations in C1, C3, C4, C5, C6, and C8. However, adjustments to C2 (Sustainable Design Methods) and C7 impact the selection of the best alternative. Specifically, when the importance of C2 exceeds 0.7, A6 relinquishes its top position to A1 (Information and communications technology). Similarly, when the importance of C7 surpasses 0.8, A10 (Warranty) emerges as the optimal choice.

## 6 Conclusion and discussions

Sustainable development stands as the prevailing trajectory within the manufacturing industry. Simultaneously, companies are actively pursuing agile methods to achieve sustainability throughout the product life cycle. This is intricately linked to the speed of product development and the competitiveness of products in the market. The initial stride towards achieving this objective involves a meticulous assessment of both sustainability and agility levels of any manufactured product within the company. In this study, WFAM (Weighted Fuzzy Assessment Method) is introduced, accompanied by a case study to demonstrate the efficacy of this proposed methodology. A significant advantage of this approach is its incorporation of human perception across all stages, enhancing its accuracy. By employing FAHP (Fuzzy Analytic Hierarchy Process) for weighting selected elements and sub-elements, utilizing a fuzzy evaluation procedure for scoring, and integrating expert advice for each aspect of sustainability and agility, the proposed approach is notably more precise.

The modification in the sustainability element of the material leads to a favorable impact on the score, yet it introduces alterations in product performance and safety. Additionally, the heightened formalization of the process aids in the punctual completion of new product development. However, this change leads to a decrease in customer orientation and product family architecture scores due to the potential adverse effects of excessive information on designers' decision-making during the product development process, adding complexity. The increased flexibility resulting from reduced Research and Development (R&D) complexity through collaboration inside of organisation represents a notable improvement, escalating from 0.25 to 0.30. Overall, a comparison between the two total sustainability indices and an agility index reveals a slight uptick in the overall sustainability index from 0.57 to 0.61 and a marginal increase in the overall agility index from 0.52 to 0.56 subsequent to altering the appearance material. This positive trend manifests across various facets of product design and the development process, illustrating some carried-over enhancements.

To highlight the accuracy of the suggested WFAM approach, a comparison was made without integrating the weights. The total sustainability index and agility index were computed independently of the weights. When contrasting these outcomes with the results obtained using the current WFAM methodology, it becomes apparent that while the values of 0.61 and 0.56 fall within the same medium range as the weighted results, disregarding weights might overlook certain subtleties. The omission of weighting or expert input could potentially result in misleading decisions. As a result, WFAM furnishes managers and decision-makers with a more precise understanding of the actual sustainability factors at play.

This research introduces a validated methodology intended to function as a guide for manufacturers in crafting more sustainable products. It showcases how minor enhancements in product sustainability and agility can positively impact sustainable manufacturing. However, this study still has several limitations. Firstly, this approach requires enterprise managers to have a strong vision for the sustainability of the product during the planning phase of product development and to be willing to adopt and apply strategies that can enhance organizational agility. Secondly, the evaluation process of this method is completed through complex calculations, which will hinder the application and promotion of this method by manufacturing enterprises. Thirdly, this method focuses on the sustainability and agility of the product development process, but there are still shortcomings in achieving the sustainability goals of the entire product lifecycle. For future endeavors, a hybrid approach could be suggested to evaluate sustainability and agility across manufacturing processes and product services, encompassing the entirety of the product life cycle.

## Data availability statement

All relevant data are within the paper.

## Funding statement

The author(s) received no specific funding for this work.

## Additional information

No additional information is available for this paper.

## CRediT authorship contribution statement

**Zhao Zhining:** Writing – original draft, Visualization, Validation, Software, Resources, Methodology, Investigation, Formal analysis, Data curation, Conceptualization. **Hassan Alli:** Writing – review & editing, Supervision. **Masoud Ahmadipour:** Writing – review & editing, Software, Methodology, Data curation, Conceptualization. **Rosalam Che me:** Supervision, Project administration.

## Declaration of competing interest

The authors declare that they have no known competing financial interests or personal relationships that could have appeared to influence the work reported in this paper.
